# The Kidscore^TM^ D5 algorithm as an additional tool to morphological assessment and PGT-A in embryo selection: a time-lapse study

**DOI:** 10.5935/1518-0557.20190054

**Published:** 2020

**Authors:** Eduardo Gazzo, Fernando Peña, Federico Valdéz, Arturo Chung, Claudio Bonomini, Mario Ascenzo, Marcelo Velit, Ernesto Escudero

**Affiliations:** 1 INMATER Fertility Clinic, Lima, Peru; 2 GENOMICS PERU, Lima, Peru

**Keywords:** time-lapse monitoring, KIDscore D5, euploidy, single embryo transfer

## Abstract

**Objective:**

To evaluate the use of implantation data algorithm *KIDscore^TM^ D5* (Vitrolife^®^, Canada) as an additional tool for morphological assessment and preimplantation genetic testing for aneuploidies (PGT-A) to improve implantation and ongoing pregnancy rates.

**Materials and Methods:**

This study looked into 912 embryos from 270 patients who underwent IVF at the INMATER Fertility Clinic in Lima, Peru, between October 2016 and June 2018. All embryos were cultured for up to five or six days in an Embryoscope^®^ time-lapse incubator (*Vitrolife^®^, Canada)* and evaluated based on the *KIDscore^TM^ D5* algorithm (KS5). Biopsies for PGT-A screening were performed in 778 (85.31%) embryos. A total of 184 single embryo transfers (68% of patients) were performed during the study period and the embryos transferred were divided into four groups: 1) euploid embryos transferred without consideration to their KS5 scores (n=86); 2) euploid embryos transferred considering their KS5 scores (n=48); 3) embryos transferred without consideration to their KS5 scores and that were not evaluated by PGT-A (n=40); and 4) embryos transferred considering their KS5 scores and that were not evaluated by PGT-A (n=10). Implantation and ongoing pregnancy rates were compared between the groups and between euploid embryos with the highest KS5 scores (KS5=6, n=25) and euploid embryos with the lowest KS5 scores (KS5=1, n=51). The correlations between KS5 scores and embryo euploidy rates were also evaluated.

**Results:**

Euploid embryo transfers in which KS5 scores were considered in the selection process had significantly higher Implantation and ongoing pregnancy rates compared to euploid embryo transfers in which selection was based on morphology (75.00% *vs.* 50.00%; *p*=0.002 and 66.66% *vs.* 48.83%; *p*=0.037, respectively). Additionally, implantation rates were significantly higher for blastocysts with the highest KS5 score (KS5=6) compared to blastocysts with the lowest score (KS5=1) (80.00% *vs.* 49.02%; *p*=0.045). Ongoing pregnancy rates were not significantly different (72.00% *vs.* 47.06%; *p*=0.105). Euploidy rates were significantly higher in the group of embryos with KS5=6 than in the group of embryos with KS5=1 (61.88% *vs.* 48.33%; *p*=0.006).

**Conclusion:**

Embryo selection based on the KS5 algorithm score improved the implantation rates of single euploid blastocyst transfers. Furthermore, embryos with the highest KS5 score had a higher probability of being euploid and implanting.

## INTRODUCTION

One of the biggest challenges in assisted reproduction continues to be the achievement of live births from single embryo transfers. Up to now, most embryologists rely on morphological assessment for embryo selection. When indicated, preimplantation genetic testing for aneuploidies (PGT-A) is a powerful addition to identify embryos with greater chances of implanting and producing healthy newborns. The introduction of incubators with time-lapse technology such as the Embryoscope^®^, along with the development of algorithms derived from morphokinetic analysis, have helped embryologists to select embryos with higher implantation rates.

Time-lapse technology allows uninterrupted monitoring of embryos without the need to assess them outside an incubator, thus keeping culture conditions stable and controlled (Cruz *et al.*, 2011). This technology enables the identification and mapping of all embryo-related morphologic events at their exact time of occurrence (Ciray *et al*., 2014). Improved embryo monitoring prompted questions about whether morphokinetic parameters might predict implantation and how they correlated with blastocyst formation, aneuploidy, and implantation potential (Meseguer *et al*., 2011; Motato *et al*., 2016; Campbell *et al*., 2013).

Implantation data algorithm *KIDscore^TM^* processes morphokinetic parameters related to implantation derived from a large embryo database shared by 24 centers (Petersen *et al*., 2016). The biologic significance of this algorithm is remarkable, since it allows the identification of slow or fast embryo development, irregular cleavage patterns, and embryos that do not achieve optimal development on day 3 or 5 (Petersen *et al*., 2016). The KIDscore algorithm helps to differentiate between morphologically normal day 3 and day 5 embryos based on the presentation of abnormal cleavage patterns during their development.

Most predictive algorithms were developed from small databases, a limitation that casts doubts as to whether they should be used routinely by assisted reproduction laboratories (Petersen *et al*., 2016). Some authors have compared embryo evaluation algorithms to the traditional morphological assessment performed by embryologists during development and the final evaluation from a designated embryologist before embryo transfer (Storr *et al*., 2018; Adamson *et al*., 2016). Other authors recommended that every laboratory should have their own embryo selection criteria and develop independent predictive algorithms based on their own data (Yalçınkaya *et al*., 2014). On the other hand, the randomized prospective study published by Goodman *et al.* (2016) did not find statistically significant differences in the clinical outcomes of embryos transferred after assessment in a time-lapse incubator; however, the results of the study were inconclusive. Regardless of the experience reported in the literature, traditional morphological assessment by an embryologist is a predominantly subjective effort. Therefore, experiences at a single laboratory are of high value for routine clinical practice.

This study aimed to look into the implantation and ongoing pregnancy rates of embryos transferred using the KS5 as an accessory tool to morphological assessment and PGT-A in embryo selection.

## MATERIALS AND METHODS

### Ovarian stimulation

All patients underwent controlled ovarian stimulation (COS) with gonadotropins combined with a GnRH antagonist in a flexible regimen. The initial doses of gonadotropins were adjusted based on age, body mass index, and prior response to stimulation (when available), from day 2 or 3 of a spontaneous cycle or after an OC cycle. FSH/LH doses ranged from 150/75 to 300/150 IU/day, while doses of HMG varied from 150 to 300 IU/day for the first five days. Starting on day 6, the doses were adjusted when needed and follicle development was followed by ultrasound examination. A GnRh antagonist was added once the leading follicle reached at least 14mm in diameter. Final follicular maturation was triggered with hCG and/or a GnRH agonist once the leading follicle cohort reached 18mm in diameter.

### Oocyte retrieval and in vitro fertilization

Ultrasound-guided oocyte retrieval was performed 36 hours after the trigger with the patients under general anesthesia. A 17G needle (Ops Classic avec Robinet, Laboratoire CCD) was used in the procedure.

Retrieved oocytes were washed with *Global^®^ total with HEPES* (LifeGlobal, Canada) medium and cultured in *Global^®^ total for Fertilization* (LifeGlobal, Canada) medium at concentrations of 5.6% CO_2_ and 5.0% O_2_ at 37ºC. All samples were incubated in *K-Systems^®^ invi cell G210* incubators for about three hours before oocyte mechanic denudation. Denudation was performed with a glass pipette with hyaluronidase and oocytes were washed with *Global ^®^ total with HEPES* (LifeGlobal, Canada) medium. Mature oocytes were cultured for 40 minutes before microinjection.

### Embryo culture

Evaluation was performed 18-20 hours after insemination. All fertilized oocytes were first transferred to an Embryoslide^®^ (Vitrolife, Denmark) dish equilibrated the night before, and then placed in the *Embryoscope*^®^ (Vitrolife, Denmark). Each Embryoslide^®^ (Vitrolife, Denmark) dish has 12 incubation wells, each containing 20 µL of GTL medium (Vitrolife, Canada) covered with 1.8ml of mineral oil *OVOIL* (Vitrolife, Canada) to avoid evaporation of the medium. The embryos were monitored for five or six days and were only removed from the incubator on day 4, when the embryos set for PGT-A analysis underwent assisted hatching. Only embryos that reached the expanded blastocyst or hatching stage were biopsied.

### Time-lapse notes and video review

The incubator image acquisition system was pre-programmed to take pictures every 10 minutes, with a resolution of 1000 x 1000 pixels in seven focal planes distant 15 µm between each other, to ensure appropriate embryo morphology evaluation at the time of video analysis.

Immediately after the embryos were taken from the *EmbryoScope*^®^, two expert embryologists (E.G. and F.P.) reviewed the videos using the *EmbryoViewer*^®^ software.

Blastulation start time was measured from the first signs of blastocoel formation and pellucid zone thinning. Inner cellular mass and trophectoderm morphological assessment were performed according to Gardner's criteria (Gardner & Schoolcraft, 1999). The next step was to calculate the KS5 of every evaluated embryo using software *EmbryoViewer*^®^.

### Blastocyst biopsy

Embryo biopsy was performed immediately after the embryos were taken from the Embryoscope^®^ using an OLYMPUS IX73 inverted microscope, a LIKOS (Hamilton Thorne) laser, TransferMan 4r (Eppendorf) micromanipulators, and *HOLDING MPH-MED-30* (Origio) and *BIOPSY MBB-FP-M-30* (Origio) micropipettes. Laser power was set on *Validation* mode (100% *power* - pulses of 430 microseconds) and no more than four laser shots were used to separate trophectoderm cells. After biopsy, *tubing* was performed according to the recommended protocol from the genetics laboratory (*Genomics Perú)*. The embryos were kept vitrified until the results from PGT-A analysis were available.

### Preimplantation genetic testing

Of the 912 embryos evaluated with the Embryoscope^®^, 778 were biopsied for PGT-A. Preimplantation genetic testing for aneuploidy was performed by means of next generation sequencing (NGS) in a Miseq^®^ (Illumina^®^ Inc) sequencer. Complete genome amplification was performed using the Sureplex method, following manufacturer instructions. Illumina^®^ Veriseq kits were used for library preparation and molecular cytogenetic data analyses were done using the Illumina BlueFuse software.

Our associated genetics laboratory, Genomics Perú, performed all genetic testing and resulting data analysis.

### Endometrial preparation and embryo transfer

All patients underwent hormone replacement therapy in preparation for frozen embryo transfer. Seven days later, ovulation was confirmed by ultrasound examination and Leuprolide acetate 3.75 mg depot intramuscular was administered in a single dose. Then, on the second day of the next cycle, the patients were started on oral estradiol valerate 6 mg daily for approximately ten days before vaginal ultrasound for endometrial lining measurement was performed. Target thickness was 7mm. Patients with thinner endometria had their doses increased by 2 mg until the target thickness was reached.

Once the target was reached and with the PGT-A results in hand, the patients were asked to define the transfer date and to start luteal phase support six days before the selected date with vaginal micronized progesterone 400 mg BID - in other words, they were treated for five complete days before embryo transfer.

A total of 184 single embryo transfers were performed. The embryos transferred were divided into four groups: 1) euploid embryos transferred without considering their KS5 score in the selection process (n=87); 2) euploid embryos transferred considering their KS5 score (n=48); 3) embryos transferred without consideration to their KS5 scores and that were not evaluated by PGT-A (n=40); and 4) embryos transferred considering their KS5 scores and that were not evaluated by PGT-A (n=10).

### Statistical analysis

The chi-square test was used in the analysis of nonparametric proportions. Parameters following a normal distribution were treated using the analysis of variance (ANOVA) test. Results were considered statistically significant when *p*<0.05. Software *Statistical Package for the Social Sciences* 24.0 (SPSS Inc.) was used in data analysis and interpretation.

## RESULTS

A total of 184 single embryo transfers were performed. All transferred blastocysts had a KS5 score (184/184) and were assigned to one of four groups depending on the factors considered during their selection for transfer.

Group 1 included 86 euploid embryos transferred without considering their KS5 scores; 43 patients had positive β-hCG tests in this group and 42 had a gestational sac with heartbeat (implantation rate: 50.0%; ongoing pregnancy rate: 48.8%). Group 2 included 48 euploid embryos transferred considering their KS5 scores as an additional selection method to PGT-A; 36 patients had positive β-hCG tests in this group and 32 had a gestational sac with heartbeat (implantation rate: 75.0%; ongoing pregnancy rate: 66.7%) (Table 1). The analysis of Groups 1 and 2 revealed a statistically significant difference in favor of single euploid embryo transfers that considered the KS5 scores in the selection process (*p*=0.002 and *p*=0.037).

**Table 1:** Clinical results

** (n=135)**	**PGT-A**			
	**KS5 (Group 2)**	**No KS5 (Group 1)**	**TOTAL**	***p*-value**
Transferred embryos	48	86	135	
Patient mean age	29.27	31.83	30.55	
Total positive β-hCG	36	43		
Positive β-hCG (%)	** 75.0% **	** 50.0% **		**0.002 **
Gestational sac w/heartbeat	**32**	**42**		
Ongoing Pregnancy	**66.7%**	**48.8%**		**0.037**
**(n=50)**	**No PGT-A**			
** **	**KS5 (Group 4)**	**No KS5 (Group 3)**	**TOTAL**	***p*-value **
Transferred embryos	10	40	50	
Patient mean age	26.7	30.22	28.46	
Total positive β-hCG	6	18		
Positive β-hCG (%)	60.0%	45.0%		**0.396**
Gestational sac w/heartbeat	2	12		
Ongoing Pregnancy	20.0%	30.0%		**0.529**

Embryos were matched with their respective KS5 scores (1 to 6). Table 2 shows the number of euploid, transferred, and implanted embryos, and of gestational sacs in each group. Very few embryos were scored 2 or 4, so their data were not considered for analysis. A significant difference was observed between embryos scored 3 and 5 only when they were compared for euploidy rate. Similarly, a significant difference was observed between embryos scored 1 and 6 when they were compared for implantation and euploidy rates, but not when they were compared for ongoing pregnancy rates.

**Table 2:** Embryos are matched with their respective KS5 scores (1 to 6). Number of euploid, transferred, and implanted embryos, and gestational sacs in each group are shown

*Score KS5*	*Embryos*	*Euploid*	*Transfer*	*Positive β-hCG*	*Implantation rate*	*p*-value	*Gestational sac w/heart beat*	*Ongoing pregnancy rate*	*p*- value	*Euploidy rate*	*p*-value
**1**	269	130	51	25	**49.0%**	***0.045***	24	**47.1%**	***0.105***	**48.3%**	***0.006***
2	4	2	0	0	0.00%		0	0.00%		50.0%	
**3**	211	111	31	18	**58.1%**	***NS ***	18	**47.4%**	***NS ***	**52.6%**	***0.042***
4	5	3	3	2	66.7%		2	66.7%		60.0%	
**5**	115	74	29	17	**58.6%**	***NS***	15	**51.7%**	***NS ***	**64.3%**	***0.042***
**6**	223	138	25	20	**80.0%**	***0.045***	18	**72.0%**	***0.105***	**61.9%**	***0.006***

**Figure 1:**
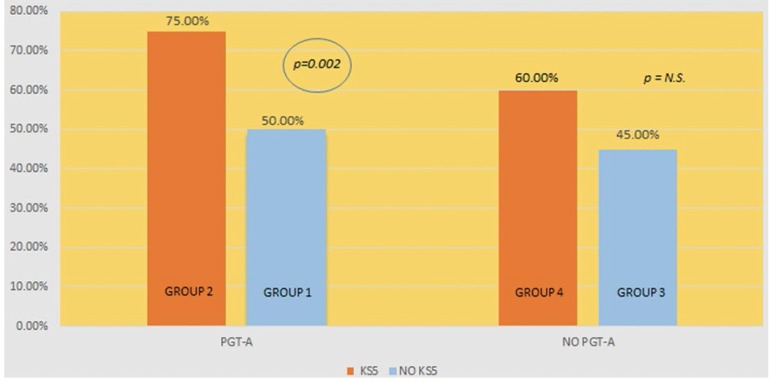
Implantation rate (with and without PGT-A) *vs*. embryo selection (with and without KS5 selection)

Group 3 included 40 embryos transferred without PGT-A analysis or consideration to KS5 scores; 18 patients had positive β-hCG tests in this group and 12 had a gestational sac with heartbeat (implantation rate: 45.0%; ongoing pregnancy rate: 30.0%). Group 4 included 10 embryos transferred considering their KS5 scores without PGT-A analysis; six patients had positive β-hCG tests in this group and two had a gestational sac with heartbeat (implantation rate: 60.00%; ongoing pregnancy rate: 20.00%). No statistically significant difference was found between implantation rates when the two groups were compared (*p*=0.396 and *p*=0.529, respectively) (Figure 1).

**Figure 2:**
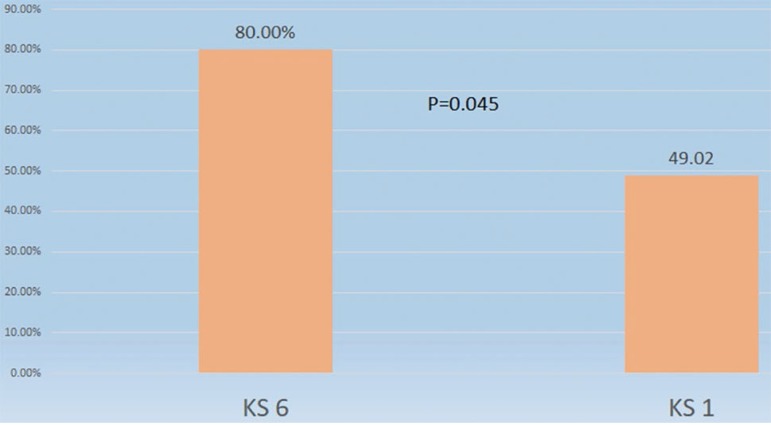
Implantation rate of euploid embryos with scores KIDscore = 6 and KIDscore = 1

Euploid embryos with the highest KS5 scores (KS5=6, n=25) had significantly higher implantation rates than euploid embryos with the lowest scores (KS5=1, n=51) (80.0% *vs.* 49.0%; *p*=0.045) (Figure 2). Ongoing pregnancy rates were not statistically different (72.00% *vs.* 47.06%; *p*=0.105)

The euploidy rate of embryos with a score of 6 was significantly higher than the rate seen in embryos given a score of 1 (61.88% *vs.* 48.3%; *p*=0.006) (Table 3).

**Table 3:** Comparison between embryos assigned a KS5 score of 6 vs. embryos assigned a KS5 score of 1

** **	**KS5 = 6**	**KS5 = 1 **	
** **	**n**	**n**	***p*-value **
Transferred embryos	25	51	-
Patient mean age	30.46	31.64	-
Total positive β-hCG	20	25	-
Positive β-hCG (%)	80.0%	49.0%	**0.045**
Gestational sac w/heartbeat	18	24	
Ongoing pregnancy rate	72.0%	47.1%	**0.105**
**EUPLOIDY RATE**	**n**	**n**	***P*-value **
Analyzed embryos	223	269	
Patient mean age	29.72	31.94	
Total Euploid embryos	138	130	
Euploidy rate (%)	61.9%	48.3%	**0.006**

## DISCUSSION

Our results confirmed the predictive properties of the KS5 algorithm for blastocysts and the use of PGT-A in producing significantly higher implantation rates for embryos selected according to their scores. Although Adamson *et al*. (2016) also described favorable results from the combination of time-lapse embryo evaluation and traditional morphological assessment, their study was performed with cleavage stage embryos, when the current trend in assisted reproduction laboratories is to transfer blastocyst stage embryos (day 5).

Yang *et al.* in 2014 showed that continued embryo monitoring by time-lapse combined with chromosome screening by array comparative genomic hybridization (aCGH) significantly improved blastocyst implantation rates. However, the sensitivity of aCGH differs from the sensitivity of the method used in our study. Next generation sequencing allows the differentiation of mosaic embryos - known for lower implantation rates - that would have been reported as euploid embryos in aCGH (Werlin *et al.*, 2017).

When assessing morphology, Basile *et al*. (2014a) found that certain morphogenetic parameters such as embryo division rates in the cleavage stage or the duration of cell cycles correlated with chromosome abnormalities, suggesting that these events might be considered as specific markers for the validation of predictive embryo selection algorithms. In the present study, higher KS5 scores also correlated with higher euploidy rates. However, neither KS5 algorithm scores nor morphokinetic parameters fully predicted embryo ploidy, leaving PGT-A as the gold standard for determining embryo ploidy status (Rienzi *et al.*, 2015).

Regarding the intrinsic analysis of the KS5 algorithm, significant differences in implantation and embryo euploidy rates were found only when extreme algorithm scores were compared - i.e., scores of 6 and 1. Basile *et al*. (2014b) performed the same analysis and found significant differences between all algorithm scores.

The lack of significant differences between consecutive categories of the KS5 algorithm may be explained, at least in part, by the irregular number of embryos in each group. Yet, the KS5 algorithm score proved useful in the selection of embryos assigned different scores in cohorts originated from the same patient, and might be used as an additional tool in morphologic assessment to improve implantation rates (Adamson *et al*., 2016). Taking the differences mentioned above into consideration, our data showed that embryos with a KS5 score of 6 are more likely to be euploid and, consequently, to yield higher implantation and pregnancy rates.

Our study also demonstrated that embryo selection using Embryoscope^®^ time-lapse monitoring and the KS5 algorithm score improved the implantation rates of euploid embryo transfers (75.00% *vs.* 50.00%). This statistically significant difference is clinically relevant when counseling couples undergoing IVF, a setting in which the use of additional tools to select euploid embryos is amply justified. Numerically higher implantation rates (60.00% *vs.* 45.00%; *p*=0.396) were observed when embryos with unknown ploidy statuses selected based on their KS5 scores were transferred, although the difference was not statistically significant. This was most probably due to the small size of the sample of embryos without PGT-A analysis.

As shown in this study, euploid embryos with better KS5 scores had significantly better implantation rates (KS5 score of 6: 80.00%; KS5 score of 1: 49.02%; *p*=0.045). Therefore, the timing of embryo cleavage might be a better predictor of implantation capacity than the final grade assigned to an embryo via morphological assessment. This was observed when two morphologically good euploid embryos had opposite KS5 scores. Predictive algorithms such as the KS5 have gained relevance in the selection of the best embryos for transfer, particularly in clinics targeting single embryo transfers as a means of eliminating multiple pregnancies. Although it cannot improve pregnancy rates, genetic screening of embryos has also become important for decreasing the probability of miscarriages and shortening the time to a live birth, the ultimate goal of assisted reproduction.

Our highly encouraging results showed the advantages offered by time-lapse technology in assisted reproduction. We firmly believe that predictive algorithms should be used as an accessory tool to traditional morphological assessment and PGT-A and incorporated into the protocols of assisted reproduction laboratories. An easy-to-implement method, predictive algorithms use basic events in embryo development observed by an embryologist as input to produce a score with which the best embryos can be selected for transfer.

## References

[r1] Adamson GD, Abusief ME, Palao L, Witmer J, Palao LM, Gvakharia M (2016). Improved implantation rates of day 3 embryo transfer with the use of an automated time-lapse-enabled test to aid in embryo selection. Fertil Steril.

[r2] Basile N, Nogales Mdel C, Bronet F, Florensa M, Riqueiros M, Rodrigo L, García-Velasco J, Meseguer M (2014). Increasing the probability of selecting chromosomally normal embryos by time-lapse morphokinetics analysis. Fertil Steril.

[r3] Basile N, Vime P, Florensa M, Aparicio Ruiz B, García Velasco JA, Remohí J, Meseguer M (2014). The use of morphokinetics as a predictor of implantation: a multicentric study to define and validate an algorithm for embryo selection. Hum Reprod.

[r4] Campbell A, Fishel S, Bowman N, Duffy S, Sedler M, Thornton S (2013). Retrospective analysis of outcomes after IVF using an aneuploidy risk model derived from time-lapse imaging without PGS. Reprod Biomed Online.

[r5] Ciray HN, Campbell A, Agerholm IE, Aguilar J, Chamayou S, Esbert M, Sayed S (2014). Time-Lapse User Group. Proposed guidelines on the nomenclature and annotation of dynamic human embryo monitoring by a time-lapse user group. Hum Reprod.

[r6] Cruz M, Gadea B, Garrido N, Pedersen KS, Martínez M, Pérez-Cano I, Muñoz M, Meseguer M (2011). Embryo quality, blastocyst and ongoing pregnancy rates in oocyte donation patients whose embryos were monitored by time-lapse imaging. J Assist Reprod Genet.

[r7] Gardner DK, Schoolcraf WB, Jansen R, Mortimer D (1999). In vitro culture of human blastocyst. Towards Reproductive Certainty: Infertility and Genetics Beyond.

[r8] Goodman LR, Goldberg J, Falcone T, Austin C, Desai N (2016). Does the addition of time-lapse morphokinetics in the selection of embryos for transfer improve pregnancy rates? A randomized controlled trial. Fertil Steril.

[r9] Meseguer M, Herrero J, Tejera A, Hilligsøe KM, Ramsing NB, Remohí J (2011). The use of morphokinetics as a predictor of embryo implantation. Hum Reprod.

[r10] Motato Y, de los Santos MJ, Escriba MJ, Ruiz BA, Remohí J, Meseguer M (2016). Morphokinetic analysis and embryonic prediction for blastocyst formation through an integrated time-lapse system. Fertil Steril.

[r11] Petersen BM, Boel M, Montag M, Gardner DK (2016). Development of a generally applicable morphokinetic algorithm capable of predicting the implantation potential of embryos transferred on Day 3. Hum Reprod.

[r12] Rienzi L, Capalbo A, Stoppa M, Romano S, Maggiulli R, Albricci L, Scarica C, Farcomeni A, Vajta G, Ubaldi FM (2015). No evidence of association between blastocyst aneuploidy and morphokinetic assessment in a selected population of poor-prognosis patients: a longitudinal cohort study. Reprod Biomed Online.

[r13] Storr A, Venetis C, Cooke S, Kilani S, Ledger W (2018). Time-lapse algorithms and morphological selection of day-5 embryos for transfer: a preclinical validation study. Fertil Steril.

[r14] Yalçınkaya E, Ergin EG, Calışkan E, Oztel Z, Ozay A, Ozörnek H (2014). Reproducibility of a time-lapse embryo selection model base don morphokinetic data in a sequential culture media setting. J Turk Ger Gynecol Assoc.

[r15] Yang Z, Zhang J, Salem SA, Liu X, Kuang Y, Salem RD, Liu J (2014). Selection of competent blastocysts for transfer by combining time-lapse monitoring and array CGH testing for patients undergoing preimplantation genetic screening: a prospective study with sibling oocytes. BMC Med Genomics.

[r16] Werlin LB, Emeny-Smith K, Martorana K, Nass T (2017). Next generation sequencing (NGS) vs Array CGH (ACGH) for all embryos?. Fertil Steril.

